# Bovine epididymal spermatozoa treatment for in vitro fertilization: Heparin accelerates fertilization and enables a reduction in coincubation time

**DOI:** 10.1371/journal.pone.0209692

**Published:** 2019-01-07

**Authors:** Andrielle T. M. Cunha, José O. Carvalho, Ana L. S. Guimarães, Ligiane O. Leme, Felippe M. Caixeta, João H. M. Viana, Margot A. N. Dode

**Affiliations:** 1 Institute of Biology, University of Brasília, Brasília, Brazil; 2 Veterinary Medicine Department, University of Espírito Santo, Alegre, Brazil; 3 School of Agriculture and Veterinary, University of Brasília, Brasília, Brazil; 4 Laboratory of Animal Reproduction, Embrapa Genetic Resources and Biotechnology, Brasília, Brazil; Justus Liebig Universitat Giessen, GERMANY

## Abstract

This study aimed to establish a protocol for in vitro embryo production using epididymal sperm (EP). Samples were obtained from ejaculated sperm (EJ) and the epididymis of 7 Gir bulls. First, the effect of heparin (+) on the viability, longevity (Experiment 1) and fertilization rates (Experiment 2) of the EP was evaluated. In experiment 2, a pool of EP and EJ sperm (n = 7) was coincubated with cumulus-oocyte complexes (COCs) for 0, 3, 6, 12 and 18 h, and the fertilization rate (FR) was evaluated. A third experiment was performed to test sperm treatments for IVP using the Percoll (P) or PureSperm (PS) gradients or a spTALP wash for sperm selection. Cleavage, blastocyst rate (BR) and embryo sex were evaluated. In experiment 4, embryos were produced using 6, 12, and 18 h of sperm-oocyte coincubation. The cleavage, BR, and total number and percentage of apoptotic cells were determined. Heparin affected EP viability, longevity and FR. After 6 h, 82% of the oocytes were fertilized in the EP+ group, a higher value (P<0.05) than that in the EJ (19%) and EP- (42%) groups. At 12 and 18 h, FR remained higher in the EP+ group, and a gradual increase in polyspermy was observed. The use of a P or PS gradient yielded a similar BR on D7 (54% and 52%), which was higher than the rate obtained using the washing method (37%). The embryos produced by EP and selected in a P or PS gradient resulted in a sex deviation in favor of male embryos (P>0.05). No differences (P>0.05) were observed among the groups that were coincubated for 6, 12 and 18 h with respect to embryo production, kinetics of development, total cell number and percentage of apoptotic cells. In conclusion, IVF time can be reduced to 6 h without affecting embryo production and quality. In addition, EP sperm selection can be performed by either a PS or P gradient.

## Introduction

Genetic material from animals of economic interest, wild animals or endangered species may be lost at any time by unexpected death or by acquired reproductive failure. In most of these cases, there is a loss of genetic material, as well as an economic loss. Therefore, efforts should be made to avoid the waste of that material, which can be achieved through the use of different assisted-reproduction biotechnologies. In fact, various assisted-reproduction techniques (ARTs) are currently available and are important tools to enable the storage and future use of this material.

The recovery and cryopreservation of epididymal sperm (EP) is one of these alternatives, because this process allows the preservation of male gametes and the maintenance of germplasm banks [1, 2]. These spermatozoa can be used in artificial insemination (AI) or in vitro embryo production (IVP), either by intracytoplasmic injection (ICSI) or by in vitro fertilization (IVF) [3, 4].

After leaving the testicles, spermatozoa are stored in the tail of the epididymis and at the moment of ejaculation, come in contact with the fluids secreted by the accessory glands, forming semen. These secretions contain several factors, including ions, lipids, energetic substrates, organic compounds and proteins [5, 6], that are important for the survival and transport of spermatozoa in the female reproductive tract [6]. In addition, proteins secreted in seminal plasma are important factors in membrane stability, heparin binding, the formation of the isthmus reservoir, sperm capacitation, and spermatozoa-oocyte interaction [6, 7]. Therefore, EP differ from EJ sperm, mainly because the former have not been exposed to accessory gland fluids. Additionally, EP sperm have had no contact with substances that are known to be important for the fertilization process. This lack of contact with seminal plasma can affect various aspects of the spermatozoa physiology, such as longevity, capacitation pathways and fertilizing potential.

Studies have shown that EP is more resistant than EJ and presents greater viability after refrigeration and longevity after thawing [8]. Considering these results, it can be assumed that other aspects related to sperm functionality are also different between EP and EJ. These differences, if any, need to be taken into account when EP is used in ARTs.

Several studies have reported the use of bovine sperm recovered from the epididymis tail in bovine IVP [3, 4, 9, 10, 11, 12, 13]; however, the results of bovine embryo production when this type of sperm is used are variable and inconsistent. Some reports showed a similar embryo rate [4, 9, 11, 14] and others showed a lower embryo rate [15] than those obtained with EJ. The lack of consistency in the results can be due to the preparation of EP sperm for the in vitro fertilization step, which usually is the same as that used for EJ sperm. Therefore, the possible physiological differences between EP and EJ are disregarded. In fact, studies in pigs have shown that methods of processing spermatozoa can affect the in vitro fertilization parameters [13]. The various steps involved in the IVF, such as the sperm selection process and its influence on quality and embryo sex [16, 17, 18], the presence and concentration of heparin in fertilization media [11, 15, 17] and the time necessary for sperm-oocyte coincubation [19], are well established for EJ sperm. However, in regard to the use of EP, little is known regarding the changes that the sperm experience after being exposed to the different treatments and in vitro conditions.

Therefore, we aimed to investigate how EP behave in the face of various procedures needed to perform IVF. Next, we evaluated the effect of heparin on EP sperm viability and longevity, as well as its effect on fertilization and embryo development. We additionally attempted to determine the best selection method and the coincubation time in which the majority of the oocytes would be fertilized.

## Results

### Experiment 1: Effect of heparin on viability and longevity of epididymal sperm

**Fig 1:**
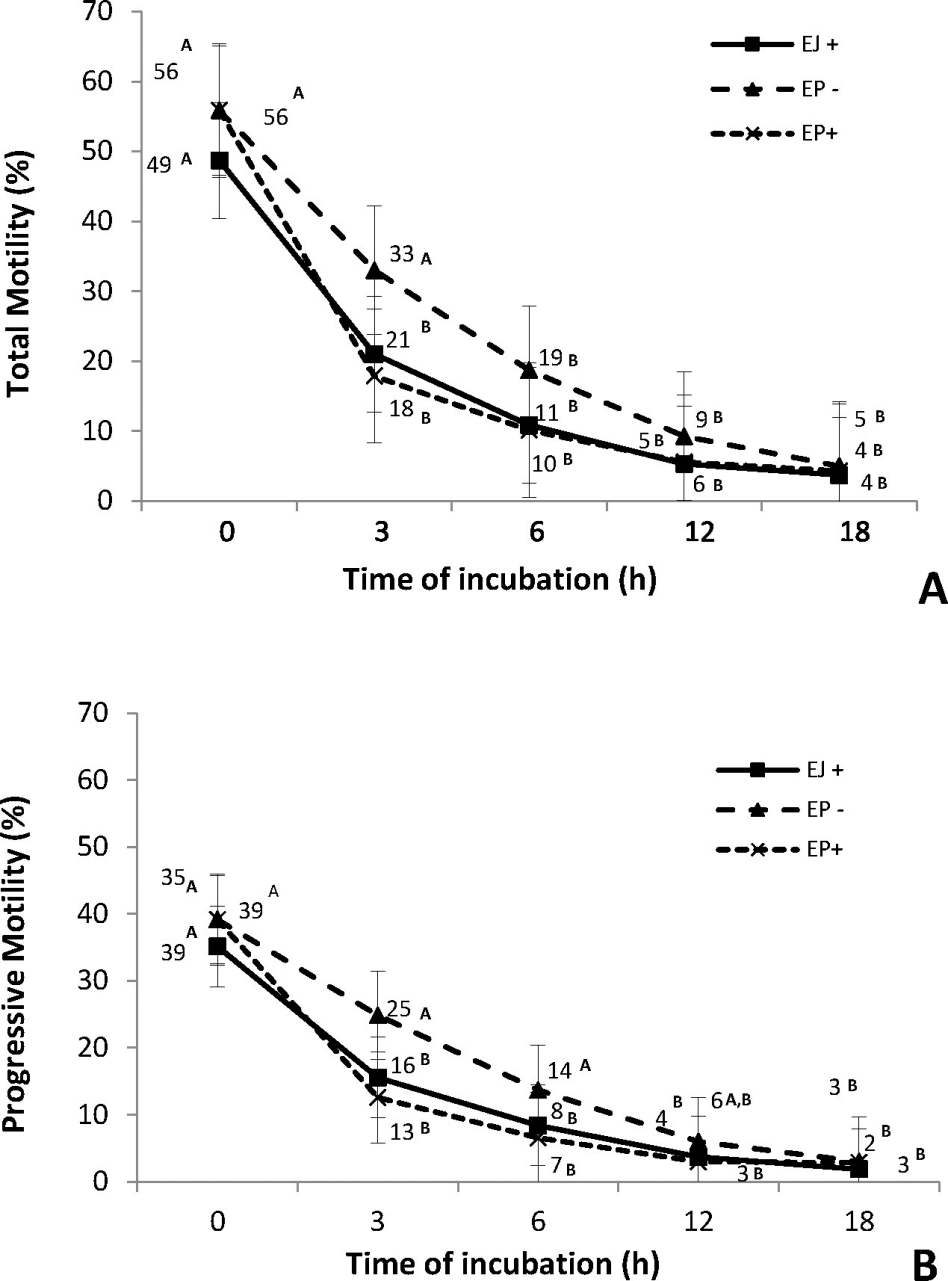
**Percentage of total (A) and progressive motility (B) at different time points of ejaculated (EJ) and epididymal (EP) sperm cultured in fertilization medium in the presence (EP+) or absence of heparin in (EP-)**. ^A, B^ Different letters in the line indicate a difference in time points (0, 3, 6, 12 and 18 hours) within each group, analyzed by Tukey-Kramer test (P≤0.05).

Total and progressive motility (Fig 1) were similar among all groups at all incubation time points. However, a decrease in total and progressive motility was observed for the EJ+ and EP+ groups after 3 h of incubation, while in the group EP-, this decrease only occurred after 6 h for total motility and after 12 h for progressive motility.

**Fig 2:**
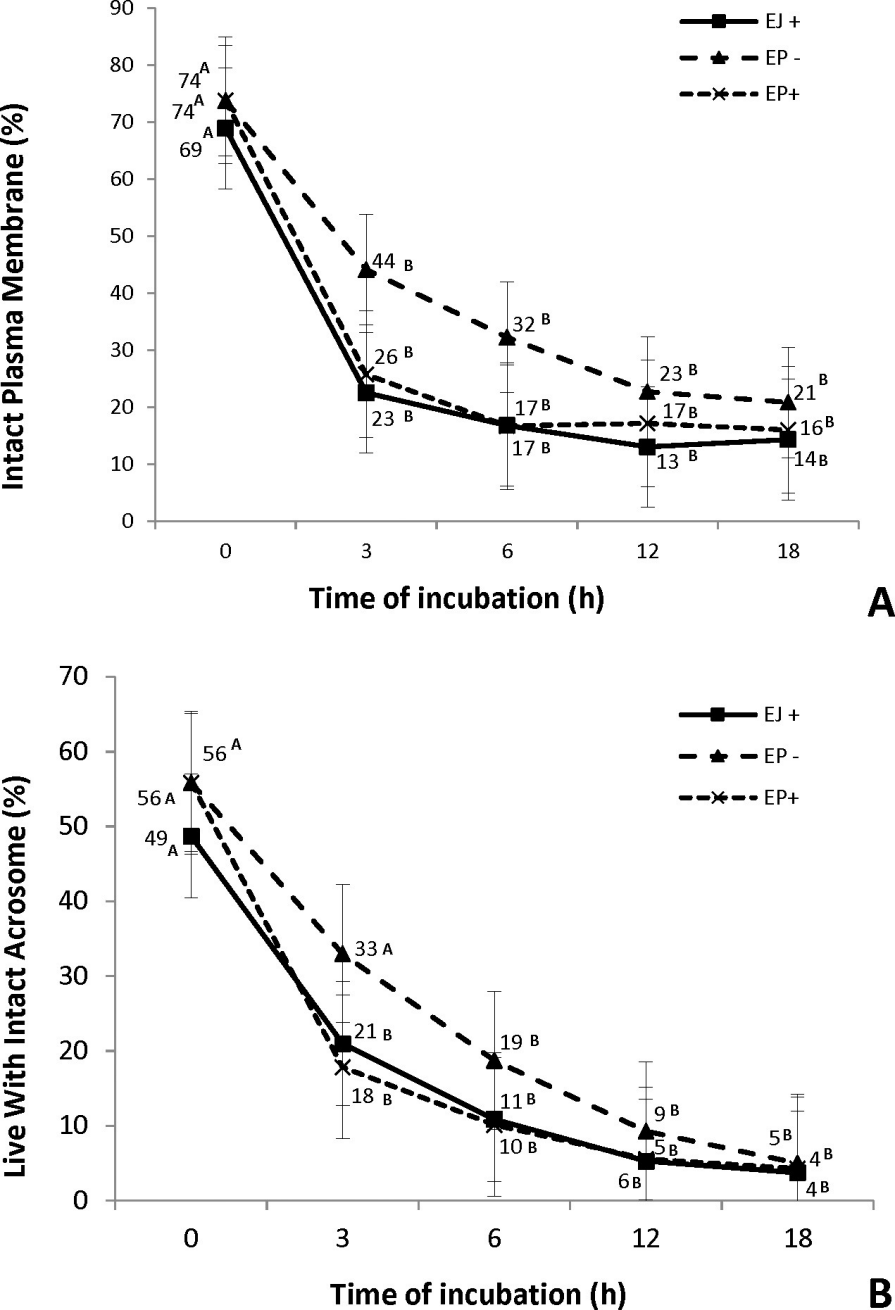
**Percentage intact plasma membrane (A) and live cells with intact acrosome (B) at different time points of ejaculated (EJ) and epididymal (EP) sperm cultured in fertilization medium in the presence (EP+) or absence of heparin in (EP-)**. ^A, B^ Different letters in the line indicate a difference in time points (0, 3, 6, 12 and 18 hours)within each group, analyzed by Tukey-Kramer test (P≤0.05).

Similar to the motility results, the percentage of cells with intact plasma membrane at the onset of incubation was similar for all groups (Fig 2). At 3 h of incubation, all groups already showed a decrease in the percentage of sperm with intact plasma membrane; however, after that time point, the proportion did not change with time. Regarding the percentage of apoptotic sperm cells, no difference was observed among the groups at any of the time points during incubation (P>0.05). Since all groups showed that less than 1% of cells were stained with the pattern characteristic of apoptotic status, we chose not to present the data.

**Fig 3:**
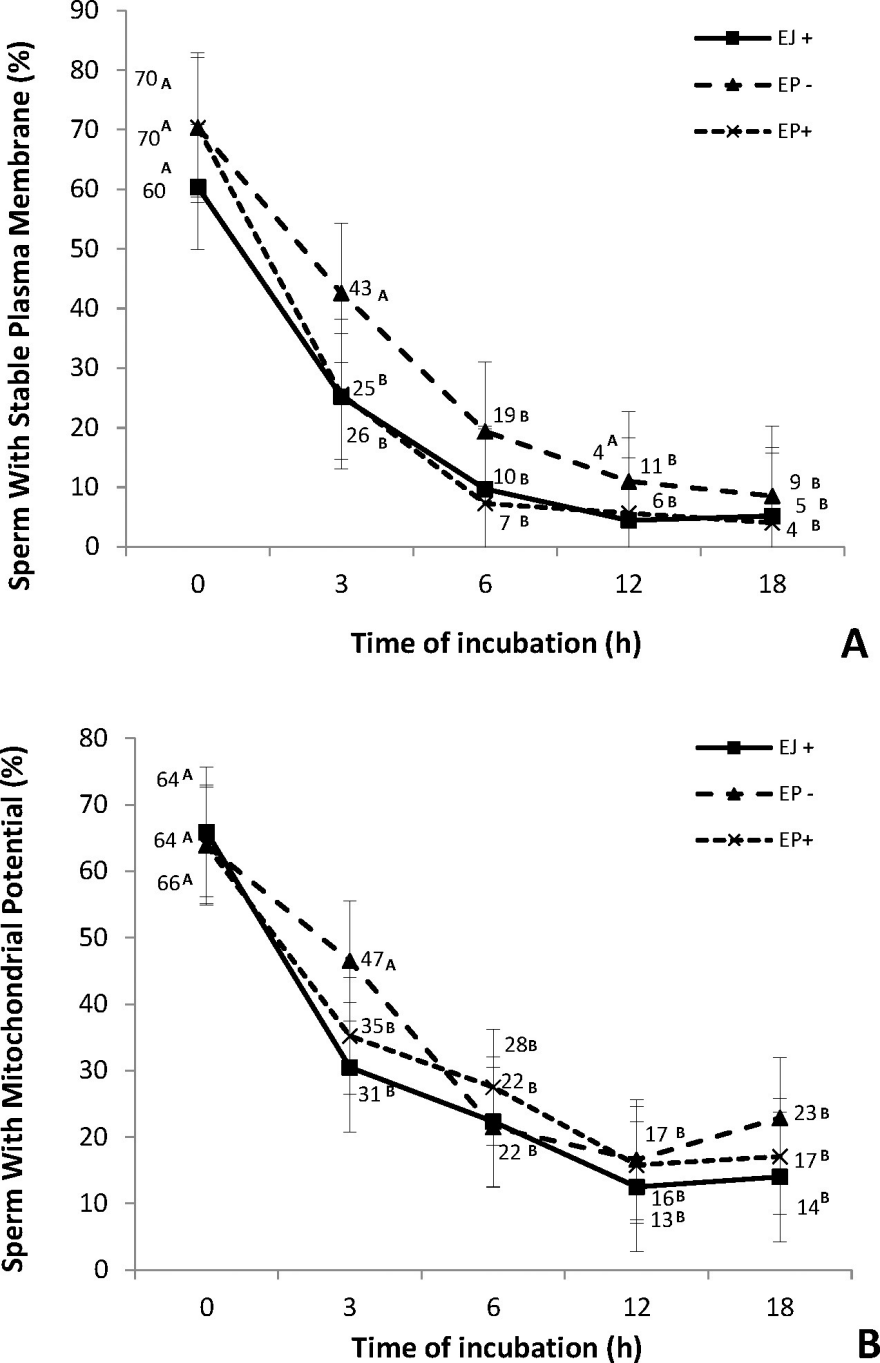
**Percentage of cells with stable plasma membrane (A) and potential mitochondrial (B) at different time points of ejaculated (EJ) and epididymal (EP) sperm cultured in fertilization medium in the presence (EP+) or absence of heparin in (EP-)**. ^A, B^ Different letters in the line indicate a difference in time points (0, 3, 6, 12 and 18 hours) within each group, analyzed by Tukey-Kramer test (P≤0.05).

The presence of heparin in the IVF medium similarly affected the mitochondrial potential (Fig 3), the acrosome integrity (Fig 2) and the plasma membrane stability (Fig 3). By 3 h of incubation, the EJ and EP+ sperm groups presented a reduction on both parameters, while in the EP-, this decrease was only evident at 6 h (Figs 2 and 3).

### Experiment 2: Effect of heparin on fertilization rates

After 3 h of sperm and oocyte coincubation, the percentage of fertilized oocytes was similar between the EP+ and EP- groups and was higher than that in the EJ group (Tab. 1). However, at 6 h of coincubation, the presence of heparin affected the ability of sperm from the epididymis to fertilize oocytes, as the epididymal-sperm group exposed to heparin showed the highest percentage of fertilized oocytes (Table 1).

**Table 1:** Fertilization rate of bovine oocytes co-incubated for 3, 6, 12 or 18 hours with ejaculated (EJ) and epididymal (EP) sperm in the absence (EP-) and presence of heparin (EP+).

Treatment	Time of evaluation	N oocytes	Fertilized	
			Total N (%)	Polyspermy N (%)
EJ+	3 h	75	3 (4.0)^a^^,^ ^A^	0 (0)^a^^,^ ^A^
EP-	3 h	58	15 (25.8)^b^^,^ ^A^	0 (0)^a^^,^ ^A^
EP+	3 h	75	23 (30.7)^b^^,^ ^A^	0 (0)^a^^,^ ^A^
EJ +	6 h	56	11 (19.6)^a^^,^ ^B^	0 (0)^a^^,^ ^A^
EP-	6 h	69	29 (42.0)^c^^,^ ^B^	0 (0)^a^^,^ ^A^
EP+	6 h	74	61 (82.4)^b^^,^ ^B^	4 (5.0)^a^^,^ ^B^^,^ ^C^
EJ +	12 h	67	45 (67.1)^a^^,^ ^C^	5 (7.0)^b^^,^ ^B^^,^ ^C^
EP -	12 h	69	53 (76.8)^a^^,^ ^b^^,^ ^C^	0 (0)^a^^,^ ^A^
EP +	12 h	70	62 (88.5)^b^^,^ ^B^	9 (12.0)^b^^,^ ^B^
EJ +	18 h	69	59 (85.5)^a^^,^ ^D^	0 (0)^a^^,^ ^A^
EP -	18 h	72	63 (87.5)^a^^,^ ^C^	2 (2.0)^a^^,^ ^C^
EP +	18 h	75	75 (100)^b^^,^ ^C^	10 (13.0)^b^^,^^B^

^a, b, c^ Values with different superscripts in the same time of evaluation are significantly different, analyzed by chi-squared test (P≤0.05).

^A, B, C, D^ Values with different superscripts in the columns indicate differences between time-points of each treatment, analyzed by chi-squared test (P≤0.05).

At 3 h of coincubation, regardless of the presence of heparin, the EP groups had higher fertilization rate than the groups receiving EJ sperm (Table 1). However, at 6 h of incubation, the effect of heparin on EP sperm was evident; the percentage of fertilized oocytes was higher than that in the EP- and EJ groups. After 12 h, the percentage of presumptive zygotes increased in the EP- and EJ groups but remained the same for the EP+ group. At 18 h of coincubation, the EJ group exhibited an increase in fertilization rate, which was similar to that observed for the EP+ group. An increase in fertilization rates was also observed at 18 h for the EP+ group. It is important to note that although the fertilization rate for this group was similar at 6 and 12 h, a gradual increase in polyspermy also occurred.

### Experiment 3: Effect of the epididymal-sperm selection method on in vitro embryo production and embryo sex

The cleavage rate was affected by sperm treatment since the EP sperm washed in spTALP showed a lower rate than the EP sperm selected by the PS gradient (Table 2). The finding of the lowest embryo production in the washed group compared to the gradient selection groups was maintained until the end of embryo development. On D6, the blastocyst rate was higher when the epididymal sperm was selected by PS (Table 2).

**Table 2:** Cleavage on D2 and blastocyst rates on days 6, 7 and 8 of culture using ejaculated sperm selected in Percoll (EJ-P), and epididymal sperm (EP) selected in Percoll (EP-P), PureSperm (EP-PS) or washed in sp-TALP (EP-spTALP). Fertilization medium of all treatments was supplemented with 10 μg/mL of heparin.

		Embryo development			
Treatment	N oocytes	Cleavage D2 N (%)	Blastocyst D6 N (%)	Blastocyst D7 N (%)	Blastocyst D8 N (%)
EJ-P	181	136 (75%)^a^^,^^b^	59 (33%)^b^	80 (44%)^b^^,^^c^	82 (45%)^a^^,^^b^
EP-P	197	157 (80%)^a^^,^^b^	73 (37%)^b^	106 (54%)^a^	110 (56%)^a^
EP-spTALP	177	127 (72%)^b^	49 (28%)^b^	66 (37%)^c^	67 (38%)^b^
EP-PS	204	164 (80%)^a^	96 (47%)^a^	107 (52%)^a^^,^^b^	110 (54%)^a^

^a, b, c^ Means followed by different letters within each column differ between groups analyzed by chi-squared test (P≤0.05).

However, this difference was not observed on D7 and D8, when the EP sperm selected using P and PS exhibited a similar blastocyst rate (Table 2). Regarding development kinetics (Table 3), in the EP group washed in spTALP, most of the embryos were in the initial blastocyst (Bi) stage. On D7, the EP group selected by PS produced more expanded blastocysts (Bx) than the EJ group selected in P. However, EP-P and EP spTALP were similar in all groups. On D8, no difference was observed in embryo development.

**Table 3:** Embryo developmental stages on days 6, 7 and 8 of culture using ejaculated sperm selected in Percoll (EJ-P) and epididymal sperm (EP) selected in Percoll (EP-P), PureSperm (EP-PS) or washed in sp-TALP (EP-spTALP). Fertilization medium of all treatments was supplemented with 10 μg/mL of heparin.

	Blastocyst D6 N (%)				Blastocyst D7 N (%)					Blastocyst D8 N (%)			
Treatment	Bi	Bl	Bx	Total embryos	Bi	Bl	Bx	Bn/Be	Total embryos	Bl	Bx	Bn/Be	Total embryos
EJ-P	22 (37%)^a^^,^^b^	20 (34%)^a^	17 (29%)^a^	59	7 (9%)^a^	25 (31%)^a^	44 (55%)^b^	4 (5%)^a^	80	15 (18%)^a^	40 (49%)^a^	27 (33%)^a^	82
EP-P	20 (27%)^b^	34 (47%)^a^	19 (26%)^a^	73	6 (6%)^a^	32 (30%)^a^	65 (61%)^a^^,^^b^	3 (3%)^a^	106	20 (18%)^a^	56 (51%)^a^	34 (31%)^a^	110
EP-spTALP	23 (47%)^a^	15 (31%)^a^	11 (22%)^a^	49	8 (12%)^a^	19 (29%)^a^	38 (58%)^a^^,^^b^	1 (2%)^a^	66	8 (12%)^a^	41 (61%)^a^	18 (27%)^a^	67
EP-PS	26 (27%)^b^	35 (36%)^a^	35 (36%)^a^	96	5 (5%)^a^	24 (22%)^a^	75 (70%)^a^	3 (3%)^a^	107	11 (10%)^a^	65 (59%)^a^	34 (31%)^a^	110

^a, b, c^ Means followed by different letters within each column differ between groups analyzed by chi-squared test (P≤0.05).

Embryo developmental stages: initial blastocyst (Bi), blastocyst (Bl), expanded blastocyst (Bx), hatching blastocyst (Bn) and hatched blastocyst (Be).

**Fig 4:**
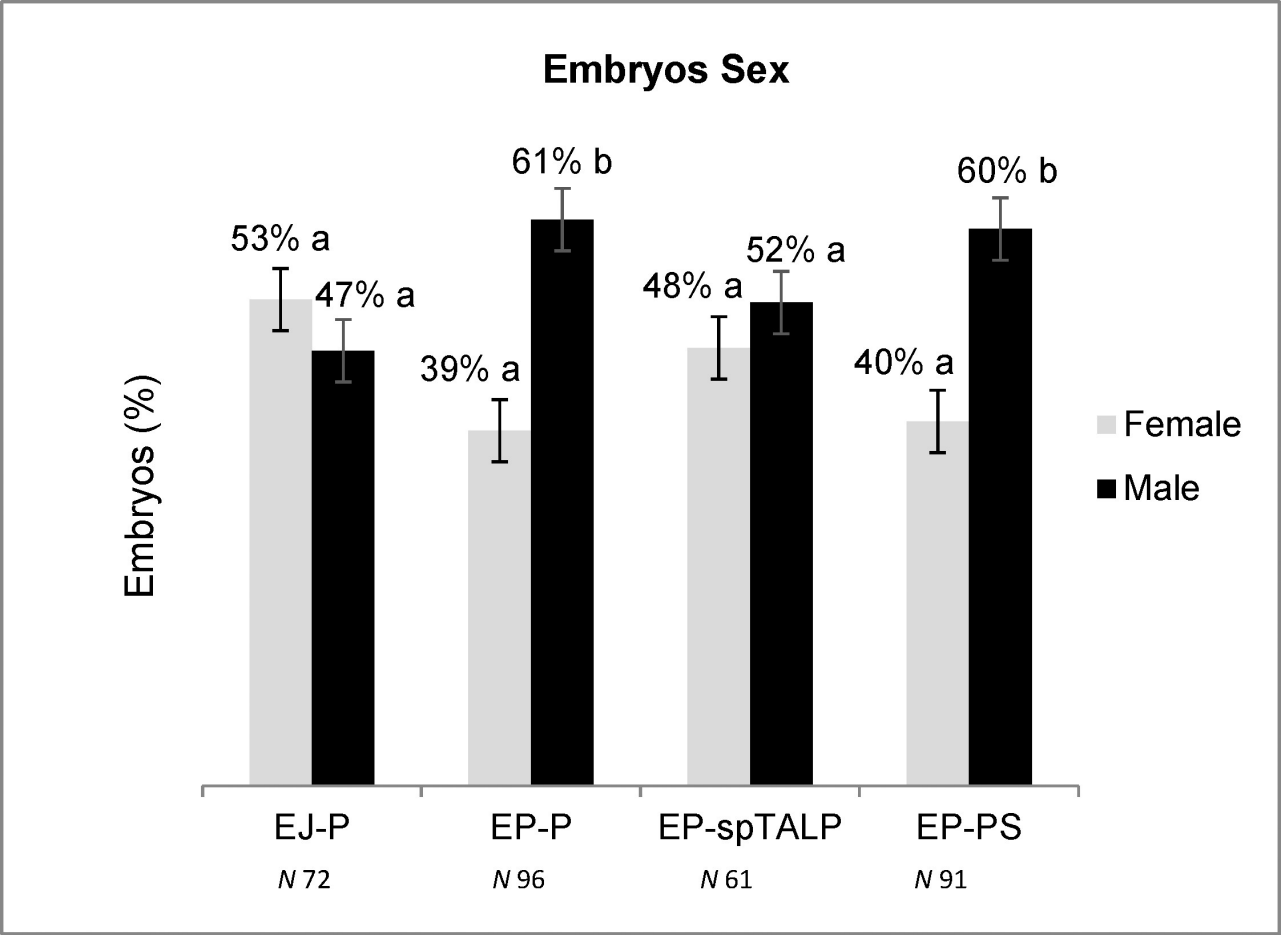
Percentage of male and female D8 embryos produced in vitro using ejaculate spermatozoa submitted to the Percoll selection (EJ-P) and epididymal sperm submitted to the Percoll (EP-P), PureSperm (EP-PS) and the wash with spTALP medium (EP-spTALP). ^a, b^ indicate difference between the proportion of male: female embryos within each group, analyzed by chi-squared test (P≤0.05).

To verify if the method used for sperm selection would affect the male:female ratio, we sexed all the embryos produced (Fig 4). The results showed that the embryos produced with the epididymal sperm selected via the 45–90% P gradient and the 40–80% PS gradient exhibited a higher number of male embryos (P<0.05).

### Experiment 4: Effect of coincubation time of EP sperm and oocyte on embryo production

To confirm that the 6 h of coincubation for IVF with EP sperm would not affect embryo production, we evaluated the embryo rate and quality. The results showed that the time of incubation affected neither the cleavage nor the blastocyst rate (Table 4) nor the embryo quality (Tables 5 and 6). The kinetics of development during embryo culture was similar among the groups (Table 5). Moreover, the number of total cells and the percentage of apoptotic cells from the expanded blastocysts did not differ between the groups (Table 6).

**Table 4:** Cleavage on D2 and blastocyst rates on days 6 and 7 using ejaculated sperm (EJ-P) or epididymal sperm (EP) co-incubated with oocytes for 6, 12 and 18 h in fertilization medium supplemented with 10 μg/mL of heparin.

		Embryo Development		
Treatment	N oocytes	Cleavage D2 N (%)	Blastocyst D6 N (%)	Blastocyst D7 N (%)
EJ 18h	155	121 (78%)	36 (23%)	65 (42%)
EP 6h	164	121 (74%)	40 (24%)	63 (38%)
EP 12h	153	118 (77%)	32 (21%)	61 (40%)
EP 18h	155	127 (82%)	35 (23%)	71 (46%)

Values analyzed by chi-squared test. There was no difference between treatments (P≤0.05).

**Table 5:** Blastocysts developmental stages on days 6 and 7 of culture originated from oocytes co-incubated with ejaculated sperm (EJ 18h) per 18 hours and with epididymal sperm for 6, 12 and 18 h (EP 6h, EP 12h, EP 18h). Fertilization medium of all treatments was supplemented with 10 μg/mL of heparin.

	Blastocyst D6 N (%)				Blastocyst D7 N (%)				
Treatment	Bi	Bl	Bx	Total embryos	Bi	Bl	Bx	Bn/Be	Total embryos
EJ 18h	9 (25%)	23 (64%)	4 (11%)	36	6 (9%)	26 (40%)	33 (51%)	0 (0%)	65
EP 6h	14 (35%)	20 (50%)	6 (15%)	40	3 (5%)	24 (38%)	34 (54%)	2 (3%)	63
EP 12h	13 (41%)	16 (50%)	3 (9%)	32	8 (13%)	20 (33%)	29 (48%)	4 (7%)	61
EP 18h	11 (31%)	22 (63%)	2 (6%)	35	6 (8%)	30 (42%)	34 (48%)	1 (1%)	71

Values analyzed by chi-squared test. There was no difference between treatments (P≤0.05). Embryo developmental stages: initial blastocyst (Bi), blastocyst (Bl), expanded blastocyst (Bx), hatching blastocyst (Bn) and hatched blastocyst (Be).

**Table 6:** Total cell number (mean ±SD) and percentage of apoptotic cells (mean ±SD) of day 7 embryos produced from oocytes co-incubated with ejaculated sperm for 18 hours (EJ 18h), and epididymal sperm for 6 (EP 6h), 12 (EP 12h) and 18 h (EP 18h).

Treatment	N embryos	Total cell number	Apoptotic cells N (%)
EJ 18h	29	158 ± 47	7 ± 4 (4.4%)
EP 6h	31	174 ± 45	7 ± 3 (4.0%)
EP 12h	26	164 ± 45	7 ± 4 (4.2%)
EP 18h	31	173 ± 43	7 ± 4 (4.0%)

Values analyzed by Tukey test. There was no difference between treatments (P≤0.05).

## Discussion

The recovery of EP sperm and its use in ARTs such as AI and IVP [1, 2, 3, 9, 15] has become a viable alternative to avoid the loss of genetic material of males that have accidentally died or have become infertile. However, the results obtained when these sperm cells are used in ARTs are inconsistent, which is probably due to lack of knowledge regarding their physiology and due to treating them in a similar manner as for the EJ. Therefore, aiming to establish the best protocol to use epididymal sperm in IVP, we evaluated their behavior in face of several factors that are involved in the preparation of sperm for its use in IVF.

Initially, we aimed to evaluate if the presence of heparin in the IVF medium would affect the physiological parameters of epididymal sperm. For this purpose, EP sperm was incubated for 18 h in the presence or absence of heparin, and the sperm samples were evaluated at various time points during culture. The results showed that EP presented a higher resistance during incubation and maintained total motility, progressive motility, the percentage of cells with mitochondrial potential and stable membrane for a longer time than the EJ sperm. The higher stability of EP sperm post-thawing has been previously reported in other studies [8, 11, 20]. However, the exposure of EP sperm to heparin altered their resistance, and the sperm exhibited a pattern of motility and viability similar to that observed for the EJ sperm.

One of the differences between EJ and EP is the exposure to seminal plasma, which contains various components, particularly proteins that bind to the plasma membrane and alter its permeability and stability. Therefore, the difference between the plasma-membrane compositions is possibly the factor responsible for the higher resistance of the EP sperm compared to EJ sperm.

The effect of heparin on EP-sperm resistance suggests that heparin has, through some mechanism, interacted and altered their physiology. It is well established that heparin interacts with seminal plasma proteins (BSPs) that bind to the epididymal spermatozoa when they are exposed to the seminal plasma in ejaculation [15, 21]. Heparin binding to BSPs induces loss of sperm surface components, which leads to the loss of cholesterol, phospholipids [22] and, finally, capacitation. Nevertheless, spermatozoa of the epididymis are not exposed to seminal plasma and, thus, do not have membrane-bound BSPs [22].

The absence of heparin receptors would not allow its interaction with EP; therefore, we were not expecting any effect of the heparin during the culture of epididymal sperm. Although the data clearly show that heparin induces changes in EP, the mechanisms and/or components involved in those changes are not known or described until now.

Conversely, in bovines, not only heparin but also other molecules are involved in capacitation events, such as high-density proteins, albumin and bicarbonate. Commonly, these components are present in the female reproductive tract or in an IVF medium [23]. Recent studies [24, 25] have reported the presence of other specific regions in the plasma membrane of porcine spermatozoa, in which different glycans can bind, allowing the sperm reservoir to formed in the oviduct. Taking these results into account, we can assume that heparin can interact, via some mechanism, with the EP sperm through other specific unknown regions, which could be involved in the sperm capacitation process.

Considering the effect of heparin on the viability and longevity of EP observed in the first experiment, we hypothesized that the presence of heparin would also affect the fertilization rate. The data showed that by 3 h of coincubation, regardless of the heparin presence, the EP sperm groups exhibited more fertilized oocytes than the EJ sperm groups. However, the effect of heparin on EP sperm was evident by 6 h of coincubation, when there was a considerable increase in the percentage of fertilized oocytes. This behavior was maintained until the end of the coincubation period. It is important to note that at 6 h, most of the oocytes of the EP sperm exposed to heparin were already fertilized and that additional coincubation time was not needed to obtain the maximum fertilization rate. In fact, additional coincubation time exhibited a detrimental effect on fertilization, because as the time increased, the polyspermy rates also increased. If we disregard the polyspermic oocytes, the fertilization rate obtained at 18 h was similar to that observed at 6 h.

Few studies have evaluated the effect of heparin on IVF using EP sperm. As observed in this study, Pavlok et al. [26] observed that a greater amount of fertilized structures when EP was used, including polyspermic structures. In contrast, a study by Stout [11] that evaluated the rate of fertilization of oocytes inseminated with EP sperm recovered from Holstein bulls showed that the presence of heparin did not affect the fertilization. However, it is important to point out that our fertilization data are in agreement with data from viability and longevity parameters, since it was shown in the first experiment that heparin affected the physiological characteristics of EP sperm. Thus, the greater the amount of spermatozoa available, the more rapidly fertilization occurred; if the coincubation was prolonged, an increase occurred in the rate of polyspermy.

After establishing the effects of heparin on EP sperm, the next step was to evaluate which sperm-selection procedure should be used. Next, the effect of two discontinuous gradients of selection and that of the washing procedure was evaluated on embryo production and quality. Our results showed that although the cleavage rate was similar, the blastocyst rate was higher when the sperm were selected via PS or P than when they were washed in spTALP. Our results using EP were similar to those reported by other studies that used EJ sperm selected by different gradients [17, 27, 28, 29], which demonstrated that discontinuous gradients resulted in a better sperm population compared with sperm that was only subjected to a washing method. Considering that discontinuous gradients such as P and PS are commonly used to obtain a high number of motile spermatozoa with more adequate fertilization function on EJ sperm, it could be expected that the same would occur for EP.

Considering that EP sperm presented some physiological differences from EJ sperm and that we were testing different discontinuous gradients to select the most suitable gradient to be used for IVP with EP sperm, the embryo sex ratio was also assessed. Surprisingly, a shift toward male embryos was observed when using EP sperm that were passed through P and PS gradients, which altered the sex ratio. The same results were not observed for the EJ that were selected using P and for EP that was only subjected to washing, since in both groups no difference from the expected 1:1 ratio was noted. These findings do not corroborate with that from other studies conducted by Martins et al. [3] and Vianna et al. [16] using EJ sperm, in which different discontinuous gradients have not affected the embryo sex ratio. One possibility for the contradictory results could be the effect of the sires, since effect of the bull on sex ratio has been demonstrated in several studies [22, 30, 31]. Nevertheless in the present study, the effect of an individual sire cannot be considered because the EP and EJ sperm used were obtained from the same animals, which leads to the supposition that the deviation may have resulted from the use of the discontinuous gradient. However, literature shows no evidence to support that discontinuous gradient can separate X and Y bearing sperm. Therefore, we cannot explain the sex ratio deviation observed in our results. Additional studies are needed to clarify it.

Finally, to confirm that IVF with EP can last only 6 h without compromising embryo development, we evaluated blastocyst production and quality using different times for IVF. In this experiment, we chose to use PS as the sperm selection method. The reason to do this was, first, because no differences were found in any of the studied parameters between the two gradient methods, and, second, because P has been previously described [32] as toxic, and thus the tendency is to exclude it from embryo production systems in the future. The blastocyst rate at D7, total cell number and percentage of apoptotic cells were similar if we used either 6 or 18 h of coincubation.

Therefore, the results confirmed that only 6 h are needed for IVF when EP sperm selected with PS is used. It has been shown that for EJ sperm, at least 12 h of coincubation is needed to reach the maximum fertilization rate [19], and this period can be extended to 18 h without affecting the embryonic quality or the polyspermy rate. However, to our knowledge, different coincubation times using EP sperm have not yet been evaluated, and the available studies that used EP sperm in embryo production used the same protocol that has been commonly used for EJ sperm. It is possible that differences in plasma membrane components, such as absence of seminal plasma and the coat proteins, or less quantity of cholesterol may be responsible for the difference in behavior of EP sperm in IVF procedures.

In conclusion, the presence of heparin in the IVF medium induces an acceleration in the fertilizing ability of EP sperm. Therefore, the time of coincubation during IVF can be reduced to 6 h, without affecting embryo production and quality. In addition, the highest rates of embryo production are obtained by selecting EP sperm with PS or P; however, both gradients altered the sex ratio, producing a greater quantity of male embryos.

## Materials and methods

### Chemicals and ethic committee approval of animal procedures

Unless otherwise indicated, the reagents were purchased from Sigma-Aldrich (St. Louis, MO, USA). All procedures with animals were approved by the Ethics Committee of Embrapa's Animal Genetic Resources and Biotechnology (Protocol CEUA–Cenargen 004/2013).

### Animals

Seven Gir bulls (Bos taurus indicus) aged between 36 and 40 months were selected, and used for EP and EJ sperm recovery. The animals belonged to an Embrapa’s experimental herd. During the experiment at the farm, the animals were raised in an extensive system and fed pasture (Brachiara brizantha), mineral salt and water ad libitum. Prior to the experiment, the bulls were subjected to three andrological evaluations, and only sires that showed a subjective total sperm motility ≥70% and a minimum of 70% morphologically normal sperm were used. Approximately six months after the end of the experiment animals were sold in an internal auction of the company.

### Sperm collection and cryopreservation

Sperm samples were collected from ejaculated and epididymal of the same animal, according to the method described by Cunha et al. [8]. Briefly, one ejaculate was collected via electroejaculation from each Gir bull. Seven to fifteen days after semen collection, all sires were orchiectomized. The testes were cleaned with saline solution (NaCl 0.9%) and 70% alcohol, and sperm collection from the cauda epididymis was performed [8]. Each epididymis was thoroughly cleaned, and the superficial blood vessels of the tail were punctured so that most of the blood could be wiped off. Next, the sperm from the epididymis tail were collected by means of cuts administered with a scalpel and removal of the white fluid coming out from the cut tubules with the aid of a blade [33].

After recovery, the EJ or EP were diluted in Tris citrate-yolk-glycerol Dilutris extender (SEMENCON–Agricultural Products Ltd., Porto Alegre, RS, Brazil), loaded at a concentration of 25 to 30 × 10^6^ sperm/straw (0.25 mL) and cryopreserved [8].

### Orchiectomy procedures

Seven to fifteen days after semen collection the bulls were castrated. First, the animals received sedation and analgesia by using xylazine (0.08 mg/kg) intravenously administered. The local anesthesia was performed using chlorhydrate of lidocaine (1000g/kg) without vasoconstrictor, into each spermatic cord and along the incision line of each testicle. A horizontal incision was made and testicles were completely exposed, then, spermatic cord was liberated from the tunica vaginalis and with a sterile lines of nylon, the spermatic cords were ligated and the surgical procedure for completely remove of testis, with the use of a scalpel, was performed. Immediately after the removal, an antiseptic solution and repellent spray were applied. After surgical procedures, animals received by intramuscular way flunixin meglumine (1.5 mg/kg) twice a day, for 3 days. A local curative was made daily, per approximately ten days once a day, or until the scrotum incision presented normal conditions of healing, without abnormal secretions.

### Sperm quality assessments

#### Motility analysis

The percentages of total and progressive motility were evaluated by the IVOS 12.3 Computer-Assisted Sperm Analyzer (CASA) system (Hamilton-Thorne Bioscience, Beverly, MA, USA). For analysis, a setup was previously adjusted for bovine spermatozoon, according to the manufacturer’s instructions. For evaluation, 8 μL of sperm was loaded into a prewarmed Makler chamber (Makler, Santa Ana, CA, USA). At least three fields were selected manually for reading and analysis.

#### Flow cytometry assessments

Evaluations were performed by flow cytometry on an AMNIS FlowSight Image Cytometer (Amnis Corp., Seattle, WA) using the INSPIRE V6.1 acquisition software. Fluorescent dyes were excited by lasers at 488 nm at 10 mW and 405 nm at 30 mW. The signals emitted by the green probes were collected in Channel 2 (505–560 nm); those emitted by the red probes were collected in Channel 4 (595–642 nm); and the blue signals were collected in Channel 7 (435–505 nm). A specific acquisition template was previously created for identifying and acquiring only sperm cells. Thus, 10.000 events were collected per sample/parameter evaluated. For analysis of the results, dot plot graphs or histograms were created from unstained control samples, and the populations were gated based on stain patterns. According to the manufacturer’s instructions and recommendations, a compensation matrix was used for all evaluations that were composed of more than one fluorescent dye, and a complete calibration of the equipment was performed each time that the system was turned off.

Plasma membrane integrity and apoptosis were evaluated using an apoptosis detection kit (Alexa Fluor 488 Annexin-V/Dead Cell Apoptosis—Molecular Probes, Eugene, OR, USA) that contained Annexin-V conjugated with Alexa Fluor 488 and Propidium Iodide (PI) solution. Sperm were classified as follows: alive (PI and Annexin-V negative), necrotic (PI positive) and apoptotic (Annexin-V positive or double positive for Annexin-V and PI).

Acrosome status was assessed using fluorescein isothiocyanate conjugated with peanut agglutinin (FITC-PNA, Invitrogen, Eugene, USA) and PI (Molecular Probes, Eugen, USA) as previously described [34], with modifications. The work solution for staining consisted of 100 μL of sodium citrate (3% diluted in NaCl 0.9%), 1 μL of PI (0.5 mg/mL), and 1.5 μL of FITC-PNA solution (1 mg/mL in PBS). PI-negative sperm were considered alive, and PI-positive sperm were considered dead. Alive or dead cells were classified as acrosome-reacted (FITC-PNA positive) or as acrosome-intact (FITC-PNA negative).

The mitochondrial membrane potential was determined using MitoTracker Green (MTGreen; Molecular Probes), as described by Celeghini et al. [35]. The work solution for staining consisted of 100 μL of sodium citrate (3% diluted in NaCl 0.9%) and 0.25 μL of MTGreen (1 mM). Sperm that were MTGreen-positive at the midpiece were considered to exhibit a positive mitochondrial potential, sperm in which MTGreen staining was absent on the midpiece were considered to be lacking a mitochondrial potential.

Stability of the plasma membrane was assessed according to the method described by Hallap et al. [36]. For this evaluation, Merocyanine 540 (M540)/YoPro1 (Molecular Probes) was used. The work solution for staining was composed of 5 mL of sodium citrate (3% diluted in NaCl 0.9%), 2.6 μL of M540 (1 mM in DMSO) and 1 μL of YoPro1 (25 μM). Cells that were M540-negative and YoPro1-positive or YoPro1-negative were considered as possessing stable sperm membranes. Cells that were M540-positive and YoPro1-positive or YoPro1-negative were considered as possessing unstable sperm membranes. To differentiate sperm cells from other events, all probes used in these assessments contained Hoechst 33342 (bisBenzimide H33342 trihydrochloride) at a concentration of 5 mg/mL (PBS), according to Hallap et al. [36].

### Assessment of fertilization and embryo production

#### Oocyte collection and in vitro maturation

Ovaries from crossbred cows (Bos indicus x Bos taurus) were collected immediately after slaughter and transported to the laboratory in saline solution (NaCl 0.9%) supplemented with penicillin G (100 IU/mL) and streptomycin sulfate (100 mg/mL) at 35°C. The cumulus-oocyte complexes (COCs) were aspirated from 3 to 8 mm diameter follicles with an 18-gauge needle; only COCs containing homogeneous cytoplasm and more than three compact layers of cumulus cells were used [37].

After selection, the COCs were transferred into a 200 μL drop of maturation medium under silicone oil. The maturation medium consisted of TCM -199 (Invitrogen, Carlsbad, CA, USA) supplemented with 10% fetal bovine serum (FBS), 0.01 IU/mL of porcine FSH, 0.1 mg/mL L-glutamine and antibiotic (amikacin, 0.075 mg/mL). The COCs were incubated for 22 to 24 h at 39°C under an atmosphere of 5% CO_2_ in air.

#### In vitro fertilization and embryo culture

Following maturation, the COCs were transferred into a drop of fertilization medium, which consisted of Tyrode’s albumin lactate pyruvate (TALP) [38] supplemented with penicillamine (2 mM), hypotaurine (1 mM) epinephrine (250 mM) and heparin (10 μg/mL). For fertilization, a pool of frozen EJ (always used as control group) or EP from the 7 bulls were used.

Sperm samples were selected by a P (Pharmacia, Freiburg, Germany) 45–90% gradient [39], PS (Nidacon Intl. AB, Gothenburg, Sweden) 40–80% gradient [9] or washing in 1 mL of spTALP medium [19]. SpTALP medium was composed of 99 mM NaCl, 3.1 mM KCl, 25 mM NaHCO_3_, 0.35 mM NaH_2_PO_4_, 10 mM HEPES, 2 mM CaCl_2_, 1.1 mM MgCl_2_, 21.6 mM sodium lactate, 1 mg/mL sodium pyruvate, 6 mg/mL BSA, and 1 mg/mL gentamicin (pH 7.4). The samples for both gradient methods were prepared in microtubes in a total volume of 800 μL [39]. After gradient selection, the samples were washed by centrifugation (300 × g per 5 min) in spTALP medium. After selection, the sperm concentration was determined using a hemocytometer, and a final concentration of 1 × 10^6^ sperm/mL was added to fertilization drops containing the COCs. Spermatozoa and oocytes were coincubated at 39°C under 5% of CO_2_ in air.

#### Fertilization rate and embryo development

To evaluate the fertilization rate, presumptive zygotes were removed from fertilization medium at 3, 6, 12 and 18 h after insemination. Next, the zygotes were denuded, fixed in acetic acid:alcohol (1:3) and stained with 1% lacmoid solution in 45% glacial acetic acid. After staining, the zygotes were examined under a phase contrast microscope (Nikon Eclipse E200, 1000X) and classified as follows: (a) non-fertilized—presence of female and absence of male chromatin or (b) fertilized—presence of female and male chromatin in the cytoplasm, as well as a decondensed sperm head, pronuclei or cleavage. Polyspermic sperm, in which the cytoplasm contains female chromatin and two or more sperm or more than two pronuclei, were included in the fertilized group.

For embryo development, after fertilization, the presumptive zygotes were washed and transferred to a 200 μL drop of SOFaaci medium [40] supplemented with 2.77 mM myo-inositol and 5% FBS. The zygotes were cultured at 39°C under 5% CO_2_ in air for 7 or 8 days. The embryos were evaluated on Day 2 (D2) postinsemination for cleavage and on Days 6 (D6), 7 (D7) and 8 (D8) for the blastocyst rate.

#### Embryo quality assessments

To evaluate the embryo quality, the parameters used were the developmental kinetics, the total cell number and the percentage of apoptotic cells. The developmental kinetics/speed of development was assessed from the stages of embryo development on D6, D7 and D8. On D8, the embryos were stored for sex analysis.

To determine the total number and percentage of apoptotic cells of D7 expanded blastocysts, a Click-iT TUNEL Alexa Fluor Thermo Fisher Kit (Waltham, MA, USA) was used. The embryos were washed in PBS supplemented with polyvinyl pyrrolidone (PVP) (1 mg/mL) and were fixed in 3.7% paraformaldehyde for 15 min. A second wash in 1 mg/mL PVP was performed, and the blastocysts were incubated in 0.5% Triton-X for 20 min. After the permeabilization procedure, the embryos were exposed to the TUNEL and Alexa Fluor 488 mix for 1 h at 37°C and treated with H33342 for 10 min. Finally, the embryos were washed in PVP and placed on slides for analysis under a fluorescence microscope. The excitation wavelength filters used for visualization were 495/519 nm for Alexa Fluor 488 and 350/461 nm for H33342. For each blastocyst, the total number of cells (blue nuclei, H33342) and apoptotic cells (green nuclei, TUNEL) were determined, and the percentage of apoptotic cells was determined.

#### Embryo sexing procedure

After culture, the embryos were stored at -20 C° in a lysis solution for sexing assessment. Sexing evaluation procedures was performed by polymerase chain reaction (PCR) using two pair of primers (Ludwing) adapted from the protocol described by Sousa et al. [41]. The first primer was specific to a region of the Y chromosome, and the second pair was specific to a bovine autosomal gene. Initially, the embryos were exposed for 5 min at 50°C in lysis solution and PCR buffer 1X that contained 15 μg of proteinase K (Invitrogen) in a final volume of 10 μL. Proteinase K inactivation was performed at 95°C for 5 min.

The PCR procedure was performed by mixing solutions containing 50 nM bovine autosomal primer and 75 nM Y chromosome primer, 200 μM dNTP, 1X PCR buffer and 1 U of Taq Polymerase Platinum (Invitrogen), to obtain a final volume of 30 μL for each individual sample. A male and female control sample, which had been previously tested and used in other assays in our laboratory, were used. The PCR program consisted of heating at 94°C for 2 min, 40 cycles of 95°C for 30 sec, 57°C for 40 sec and 72°C for 40 sec and completing primer extension at 72°C for 3 min. The products were visualized on a 1.5% agarose gel via staining with ethidium bromide (10 mg/mL) under ultraviolet light. When two amplicons were detected (280 and 210 base pairs), the embryo was identified as male, whereas detection of one amplicon (280 base pairs) indicated a female embryo.

## Experimental design

### 1. Effect of heparin on viability and longevity of epididymal sperm

To determine if supplementation of fertilization medium with heparin would affect the post-thawing longevity and viability of epididymal sperm, the EJ and EP groups were used. All bulls were used in all groups, in a factorial design. The samples of all animals were individually analyzed, and each animal was considered a biological replicate. Each sample was composed of a pool formed by four straws from each animal/group; this procedure was necessary to have a sufficient amount of cells for cytometry and CASA assessments. Straws were thawed, pooled, subjected to a P 45–90% gradient and adjusted to a final concentration of 2 × 10^6^ sperm/mL in a final volume of 400 μL of fertilization medium with or without heparin supplementation. EP and EJ sperm were distributed into three groups: 1) EJ +: EJ incubated in fertilization media; 2) EP +: EP incubated in fertilization media and 3) EP-: EP incubated in fertilization media without heparin. The samples were maintained at 39°C under a 5% CO_2_ atmosphere for 18 h.

Assessments were performed at 0 h and after 3, 6, 12 and 18 h. At each time point, a sample from each group was removed from the incubator and was used to assess total and progressive motility, acrosome integrity, apoptosis, necrosis, plasma membrane integrity, membrane stability and mitochondrial potential.

### 2. Effect of heparin on fertilization rate

To evaluate if the presence of heparin in the fertilization medium would affect the ability of EP to fertilize the oocytes, they were coincubated for different periods of time during IVF. To minimize the bull effect, EP and EJ sperm used in this experiment was obtained from pooling one straw from each of the seven bulls. After pooling the straws, motile sperm from each group (EJ and EP) were selected by centrifugation through a 45–90% discontinuous P gradient, as described above.

A total of 829 oocytes were used in a seven-replicate experiment. In each replicate, the oocytes were distributed into three treatments: 1) EJ+H: oocytes were coincubated with EJ sperm in fertilization medium supplemented with heparin; 2) EP+H: oocytes were coincubated with EP sperm in fertilization medium that was supplemented with heparin; 3) EP-H: oocytes were coincubated with EP sperm in fertilization medium without heparin. At 3, 6, 12 and 18 h postinsemination, the oocytes were denuded and fixed to assess the fertilization rate.

### 3. Effect of the epididymis-sperm selection method on in vitro embryo production and embryo sex

The aim of this experiment was to assess if the sperm-selection method could affect not only embryo production but also the sex ratio of the produced embryos. Based on the results obtained from the second experiment, heparin was included in the fertilization medium for all treatments. Three different methods of sperm preparation for IVF were evaluated. A total of 759 COCs were used in a four-replicate experiment.

Oocytes were fertilized with a pool composed of EP or EJ sperm obtained from the same seven sires used in the previous experiments. Treatments were defined as follows: 1) EJ-P: oocytes were coincubated with EJ sperm that were subjected to P gradient selection; 2) EP-P: oocytes were coincubated with EP sperm selected by a P gradient; 3) EP wash: oocytes were coincubated with EP sperm washed in spTALP medium; 4) EP-PS: oocytes were coincubated with EP sperm selected by a PS gradient. The cleavage, blastocyst rates and kinetics of embryo development were assessed at D2, D6, D7 and D8. On D8, the embryos were classified and stored until used for sexing analysis procedures.

### 4. Effect of sperm-oocyte coincubation time on embryo production

To confirm that 6 h of coincubation (experiment 2) would not affect embryo production, a total of 627 oocytes were used in a five-replicate experiment. Four treatments were used: 1) EJ 18h: oocytes were coincubated with EJ sperm for 18 h; 2) EP 6 h: oocytes were coincubated with EP sperm for 6 h; 3) EP 12 h: oocytes were coincubated with EP sperm for 12 h and 4) EP 18 h: oocytes were coincubated with EP sperm for 18 h. For all groups, the IVF medium was supplemented with heparin, and sperm selection was performed using the PS discontinuous gradient. Cleavage and blastocyst rates were assessed on D2, D6 and D7. On D7, expanded blastocysts were stored for TUNEL analysis.

### Statistical analysis

Data of sperm viability/longevity and CASA sperm motion variables were analyzed by analysis of variance (ANOVA) followed by Tukey–Kramer multiple comparison tests using SAS version 9.4 (SAS virtual University Edition). Analyses considered the main effects of group (EJ+, EP+, and EP-), time (hours 0, 3, 6, 12, and 18), and their interactions. The SAS MIXED procedure with a REPEATED statement was used to account for the autocorrelation between sequential measurements. Fertilization and embryo and sex rates were compared by the chi-squared test and TUNEL data by the Tukey test; for both analyses, the Prophet 5.0 statistical package (BBN Systems and Biotechnology, 1997, USA) was used. For all results, a level of 5% (P≤0.05) was considered significant.
